# Sex-Dependent Estimation of Spinal Loads During Static Manual Material Handling Activities—Combined *in vivo* and *in silico* Analyses

**DOI:** 10.3389/fbioe.2021.750862

**Published:** 2021-11-02

**Authors:** Ali Firouzabadi, Navid Arjmand, Fumin Pan, Thomas Zander, Hendrik Schmidt

**Affiliations:** ^1^ Julius Wolff Institute, Berlin Institute of Health at Charité—Universitätsmedizin Berlin, Berlin, Germany; ^2^ Department of Mechanical Engineering, Sharif University of Technology, Tehran, Iran

**Keywords:** manual material handling, spinal loads, trunk muscle forces, sex differences, musculoskeletal models

## Abstract

Manual material handling (MMH) is considered as one of the main contributors to low back pain. While males traditionally perform MMH tasks, recently the number of females who undertake these physically-demanding activities is also increasing. To evaluate the risk of mechanical injuries, the majority of previous studies have estimated spinal forces using different modeling approaches that mostly focus on male individuals. Notable sex-dependent differences have, however, been reported in torso muscle strength and anatomy, segmental mass distribution, as well as lifting strategy during MMH. Therefore, this study aimed to use sex-specific models to estimate lumbar spinal and muscle forces during static MHH tasks in 10 healthy males and 10 females. Motion-capture, surface electromyographic from select trunk muscles, and ground reaction force data were simultaneously collected while subjects performed twelve symmetric and asymmetric static lifting (10 kg) tasks. AnyBody Modeling System was used to develop base-models (subject-specific segmental length, muscle architecture, and kinematics data) for both sexes. For females, female-specific models were also developed by taking into account for the female’s muscle physiological cross-sectional areas, segmental mass distributions, and body fat percentage. Males showed higher absolute L5-S1 compressive and shear loads as compared to both female base-models (25.3% compressive and 14% shear) and female-specific models (41% compressive and 23.6% shear). When the predicted spine loads were normalized to subjects’ body weight, however, female base-models showed larger loads (9% compressive and 16.2% shear on average), and female-specific models showed 2.4% smaller and 9.4% larger loads than males. Females showed larger forces in oblique abdominal muscles during both symmetric and asymmetric lifting tasks, while males had larger back extensor muscle forces during symmetric lifting tasks. A stronger correlation between measured and predicted muscle activities was found in females than males. Results indicate that female-specific characteristics affect the predicted spinal loads and must be considered in musculoskeletal models. Neglecting sex-specific parameters in these models could lead to the overestimation of spinal loads in females.

## Introduction

Manual material handling (MMH) activities are regularly performed in daily life as well as in occupational workstations (Craig et al., 2015). These activities could expose the worker to external forces/moments under various postures such as trunk bending and twisting or a combination thereof. During MMH activities, trunk muscles demonstrate high levels of activation/coactivation thus imposing large loads on the spine passive structures (Zander et al., 2015; Corbeil et al., 2019). While several studies have indicated an association between MMH tasks and increased spinal loads thus identifying MMH as a risk factor for low back pain (Hoogendoorn et al., 2000; Palmer et al., 2003; Davis et al., 2005), this association remains debatable (Swain et al., 2020). The knowledge of spinal loads under various MMH conditions can therefore provide appropriate insight into the mechanism of such a likely association. An accurate estimation of trunk muscle forces and spinal loads during MMH activities is also required to design safer workplaces and effective injury prevention programs.

In an effort to gain an in-depth evaluation of spinal loads during MMH tasks, multiple *in vivo* and *in silico* methods have been used (Cruz et al., 2019; Dreischarf et al., 2016). Although *in vivo* studies provide valuable knowledge on spinal loading (Nachemson, 1981; Rohlmann et al., 2014; Wilke et al., 2001), these measurements are challenging, complex, costly and invasive. As alternatives, biomechanical models have therefore been developed to predict internal spinal loads. In this context, a number of musculoskeletal models (open-source/commercial software), regression equation, and artificial neural networks have emerged as robust and relatively accurate options (Damsgaard et al., 2006; Delp et al., 2007; Arjmand et al., 2011; Dreischarf et al., 2016; Aghazadeh et al., 2020). Electromyography (EMG)-driven (Marras and Granata, 1997; McGill and Norman, 1986), optimization-driven (Brown and Potvin, 2005; Damsgaard et al., 2006) and hybrid (EMG-Assisted Optimization) (Cholewicki and McGill, 1994; Mohammadi et al., 2015; Gagnon et al., 2016; Samadi and Arjmand, 2018) models have been used. These models predict joint loads and muscle forces through *in vivo* kinematics and/or EMG data. However, to account for the differences between individuals, models should be personalized or scaled based on individuals’ kinematics and anthropometric data.

Anybody Modeling (AB) System (Anybody^®^ Technology, Aalborg, Denmark), an optimization-driven model, is a scalable full-body model with a highly detailed musculature for the lumbar spine. This model has been used in many studies to predict spinal loads (Arshad et al., 2017; Asadi and Arjmand, 2020; Behjati and Arjmand, 2019; Ignasiak et al., 2016b; Rajaee et al., 2015; Zander et al., 2015) and could be applied to simulate a wide range of MMH activities. Spinal compressive loads predicted by the AB full-body model during different activities, including the MMH, have been validated versus *in vivo* intradiscal pressure data (Wilke et al., 2001) by several studies (Bassani et al., 2017; Ignasiak et al., 2016a; Rajaee et al., 2015; Rasmussen et al., 2009). These studies have indicated that the AB model is a robust tool for accurately evaluating spinal loads in physiological activities.

For the biomechanical risk assessment, the majority of previous studies have evaluated spinal loads while focusing on male individuals. However, notable sex-dependent kinematic differences in joint movements (Plamondon et al., 2017; Sheppard et al., 2016), lumbo-pelvic coordination (Pan et al., 2020; Pries et al., 2015), and lifting style (Haddas et al., 2015; Lindbeck and Kjellberg, 2001) have been reported. Furthermore, muscle cross-sectional areas (Anderson et al., 2012; Marras et al., 2001), body anthropometric measures, and mass distribution in upper body (Shan and Bohn, 2003; Davis et al., 2005) are also significantly different between sexes. These sex differences may influence muscle activities and spinal loads during MMH tasks (Marras et al., 2003; Plamondon et al., 2017) thereby suggesting that the previous model findings for male workers cannot be generalized to female ones. In accordance with recent greater participations of females in physically demanding jobs, epidemiological studies have reported higher work-related physical injuries (Hansen et al., 2018) and prevalence of low back pain in females than males (Wu et al., 2020).

To date, only few studies have investigated the role of sex and differences in spinal loads between males and females during common lifting tasks (Ghezelbash et al., 2016; Ghezelbash et al., 2018; Kumar, 1990; Marras et al., 2003). Marras et al. (2003), using a single level EMG-driven model without a comprehensive scaling approach, showed that males had significantly greater compression spine forces than females. Ghezelbash et al. (2016) investigated the effect of sex differences and other personalized factors (age, body height (BH), and body weight (BW)) on spinal loads using a kinematics/optimization-driven musculoskeletal trunk finite element model and found that sex has small effects on spinal loads during symmetric lifting tasks. For their model simulations, they used available kinematics data in the literature (Pries et al., 2015) that had been collected during maximal upper body flexion with no loads in hands. It has, however, been shown that lifting/holding external loads in hands influences trunk kinematic (Davis and Marras, 2000; Granata and Sanford, 2000). In addition, identical segmental mass distributions were used for both sexes despite the fact that the mass distribution of the upper body is significantly different between males and females (Davis et al., 2005).

All the sex-related differences findings in the literature and epidemiology studies highlight the urgent need to account for inherent sex differences when predicting spinal loads via biomechanical modeling approaches. Therefore, the current study aimed to predict spinal loads and trunk muscle forces during different MMH tasks, using the full-body, subject- and sex-specific models driven by *in vivo* kinematic and ground reaction data. Absolute and normalized (to BW) L5-S1 loads were compared between males and females. It was hypothesized that including sex-specific parameters into the musculoskeletal model markedly affect their predictions for spinal loads during MMH tasks.

## Materials and Methods

### Participants

Twenty healthy volunteers (10 males and 10 females) with no professional lifting experience participated in the study. Males had a significantly greater body height, weight, body mass index (BMI) (*p* < 0.05) but not age (*p* = 0.724) than females (Table 1). Participants had no history of pain in the back, pelvis, and hip in the 12 months prior to the measurements and no spinal or pelvic surgery history. The study was approved by the Ethics Committee of the Charité—Universitätsmedizin Berlin (EA1/059/21). After explaining the tests to each participant, he/she signed a written informed consent.

**TABLE 1:** Demographic data (mean ± standard deviation) of the participants. Bold values show that males have a significantly greater body height, weight, and body mass index (BMI) (*p* < 0.05) than females.

	Female	Male	*p*-value
Number of participants	10	10	-
Age (year)	31.9 ± 7.6	33.0 ± 7.4	0.724
Height (cm)	168.5 ± 3.5	176.9 ± 8.8	**0.009**
Weight (kg)	56.4 ± 4.5	77.3 ± 9.8	**<0.001**
BMI (kg/m^2^)	19.92 ± 1.9	24.7 ± 2.2	**<0.001**

### Measurement Devices

Three-dimensional motion analysis was carried out using the Vicon Motion Capturing System (Vicon Motion Systems, Inc., Oxford, United Kingdom). The system consisted of 10 high-speed infrared cameras to track retro-reflective skin markers placed over participant’s body with a sampling rate of 200 Hz. Ground reaction forces (GRFs) were measured by two floor-embedded force plates (AMTI, model OR6-6, Watertown, MA, USA) sampling at 1,000 Hz. A wireless EMG device (Delsys, Inc., Boston, MA) was used to record muscle activities at 2000 Hz. EMG and force plate data were integrated into the Vicon Nexus system and synchronized with the Vicon data.

### *In vivo* Data Collections

**FIGURE 1:**
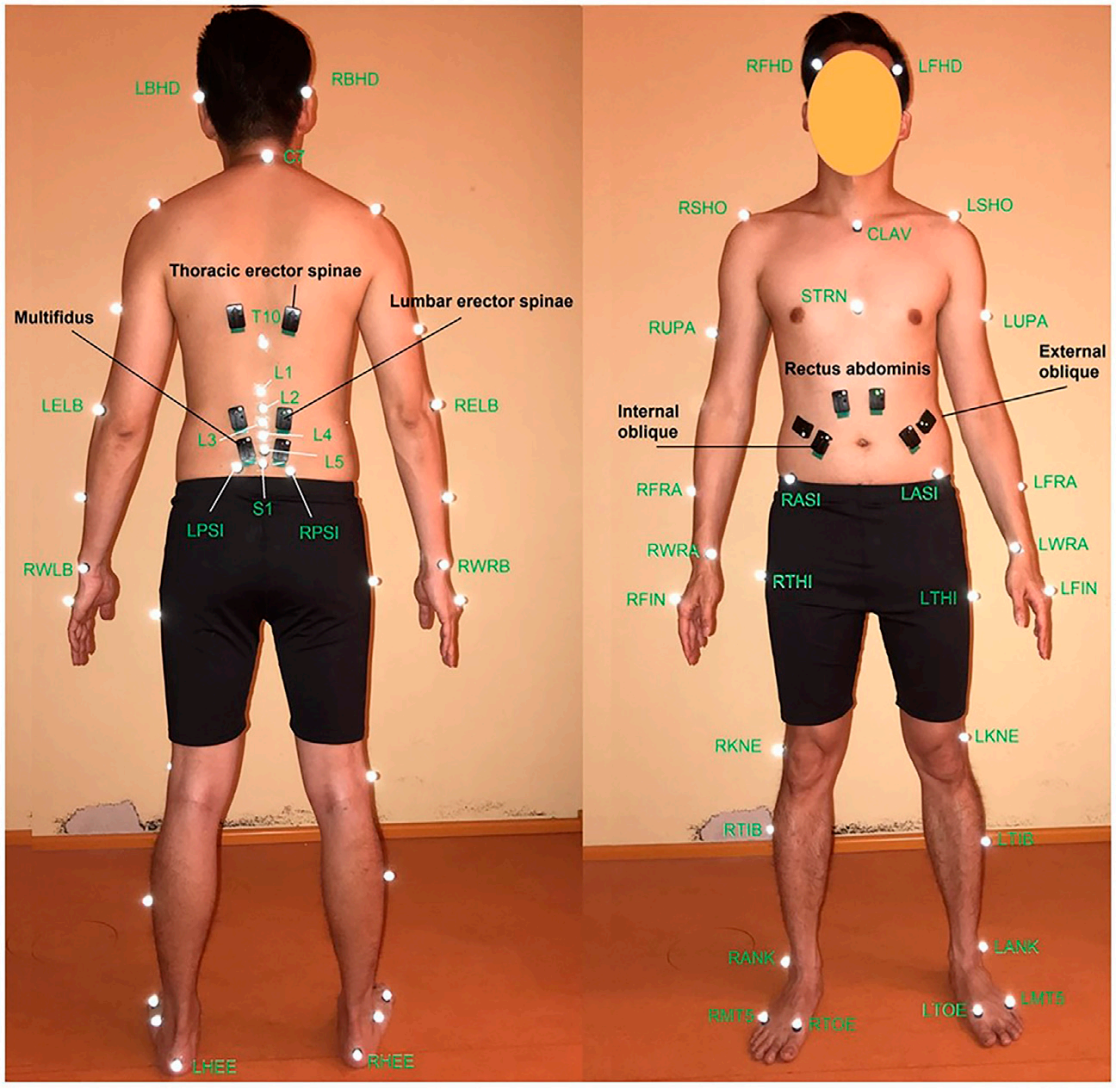
Position of VICON markers (white ones) (Arshad et al., 2017) and EMG sensors (black ones) (McGill, 1991) from **(left)** back and **(right)** front views.

A marker set consisting of 47 reflective markers (12 mm diameter) was used to capture body motion during gait and different lifting tasks. According to our previous study (Arshad et al., 2017), markers were placed on the anatomical landmarks of different body segments (head–neck, trunk, pelvis, arms, forearms, thighs, and feet) based on the Vicon Plug-in gait marker configuration. Six additional markers were placed on the superior spinal process of the lumbar vertebrae and the sacrum (Figure 1). Twelve surface EMG electrodes recorded trunk muscle activities. Electrodes were positioned bilaterally on six superficial back and abdominal muscles as follows (McGill, 1991): multifidus (∼2 cm lateral to midline at the L5), lumbar erector spinae (∼3 cm lateral to midline at the L3), thoracic erector spinae (∼5 cm lateral to midline at the T9), external oblique (∼10 cm lateral to midline above umbilicus and aligned with muscle fibers), internal oblique (below to the external oblique sensors and superior to the inguinal ligament), and rectus abdominis (∼3 cm lateral to midline above the umbilicus) (Figure 1). Participants performed 3 trails of Maximal Voluntary Contractions (MVC) for back and abdominal muscles (Konard, 2006). During MVC measurements, subjects were verbally encouraged to exert their maximum efforts. Their hands were held crossed on the chest while the hip and legs were fully constrained.

To prepare the kinematics data for model simulations, motion capture data were pre-processed in Vicon Nexus 2.8.1 (Vicon Motion System, Oxford Metrics Inc., Oxford, UK) for marker labeling and gap-filling. Missing or occluded markers were reconstructed *via* the spline fill, pattern fill, or rigid body fill algorithms (Vicon Nexus 2, 2018). A zero-lag 2nd order low-pass Butterworth filter was used with the cut-off frequency of 6 Hz for trajectories of the reflective markers, and the cut-off frequency of 20 Hz for measured GRFs. A band-pass filter (30–450 Hz) was applied to the EMG signals to reduce the effect of artifacts and noises. Subsequently, the signals were rectified, low-pass filtered (cut off frequency 3 Hz), and normalized relative to their MVC peak values.

### Tasks

**FIGURE 2:**
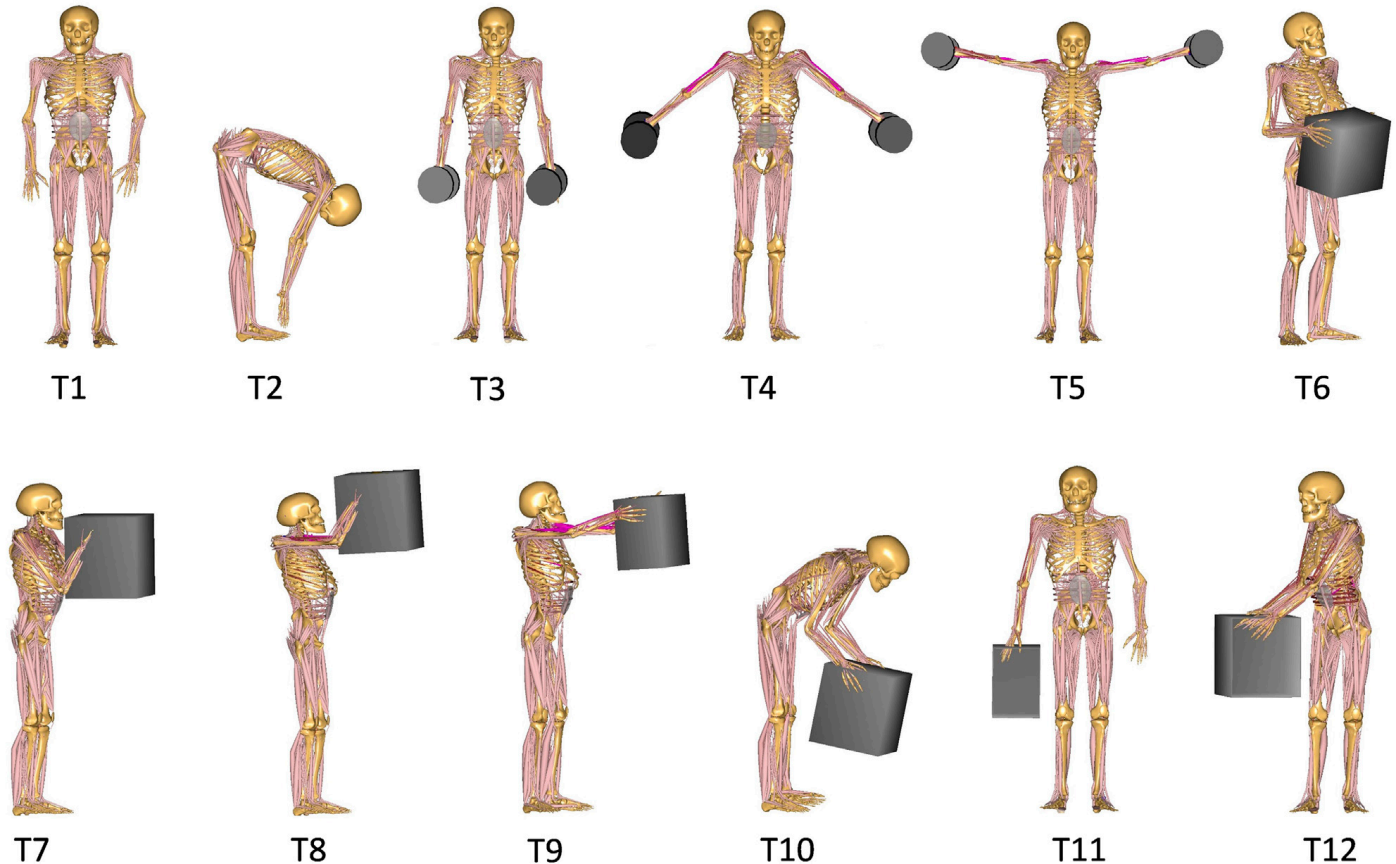
Different lifting tasks performed by participants and simulated by the model.

Participants performed a dynamic lifting task that started from the moment they touched the weight, lifted it, held it in the final position for 3 s and finally ended with putting it back on the ground. For our analyses here, we only considered the 3 s of static holding of the weight (Figure 2). They performed a total of twelve symmetric and asymmetric static load-handling tasks in a randomized order (Figure 2). These tasks have been selected so that different parameters that might influence spinal loads, such as postures, lifting techniques, horizontal distance of the hand load, and lifting height could be included in the analyses (Rajaee et al., 2015). The end (static) position of each lifting task differs as follows:

Reference Postures:

➢Task 1 (T1): relaxed upright standing
➢Task 2 (T2): full upper body flexion with straight knees and without loads in hand

Symmetric holding of two 5 kg dumbbells on each side of the body with:

➢ Task 3 (T3): arms close to the trunk at the thigh height
➢ Task 4 (T4): arms abducted 45° at the hip height
➢ Task 5 (T5): arms abducted 90° at the shoulder height

Symmetric holding of a 10 kg box in front of and close to the body at the:

➢ Task 6 (T6): hip height
➢ Task 7 (T7): chest height
➢ Task 8 (T8): head height

Symmetric holding of a 10 kg box in front of the body:

➢ Task 9 (T9): at the chest height with extended elbows
➢ Task 10 (T10): with flexed back and extended knee

Asymmetric holding of a 10 kg box:

➢ Task 11 (T11): by one hand in the favored side
➢ Task 12 (T12): in front of the body and twisting the trunk to the right side

### Musculoskeletal Model

#### Base Models

In this study, a commercially available MoCap-FullBody musculoskeletal model from the AnyBody Managed Model Repository v.1.6.2 of the AnyBody Modeling System software v. 6.0.4 (AnyBody Technology A/S, Aalborg, Denmark) was used as the base-model. This validated model for males (Bassani et al., 2017) included the Twente Lower Extremity Model (TLEM) (Klein Horsman et al., 2007) and a detailed lumbar spine model (Zee et al., 2007). The spine model consisted of 7 rigid-bodies, including the pelvis, lumbar, and a rigid thoracic segment. In the lumbar part, each vertebra was modeled as a rigid segment with 3-DoF spherical joints in between. All significant muscles related to the trunk, arms and legs were included in the model. A total of 188 muscle fascicles were used to represent the muscular architecture of the lumbar spine model. Trunk muscles were grouped as global (attached to thoracic spine) and local (attached to lumbar spine) (El-Rich et al., 2004). Intervertebral joint stiffnesses were considered as linear in flexion, extension, lateral bending, and axial rotation. Intra-abdominal pressure was modeled as an abdominal volume wrapped by the transverse muscles with the maximum upper bound limitation of 26.6 kPa (Essendrop, 2004). During body movements, these muscles acted on the abdominal volume, and due to the change in the volume, the intra-abdominal pressure was generated. The spine curvature was adjusted based on the markers on the hip and thorax. Intersegmental lumbar rotations (lumbar spine movement rhythm) were prescribed as a function of the 3D angle between pelvis and trunk. This lumbar spine rhythm was taken from (White and Panjabi, 1990), which provides the representative rotation of each lumbar joint in flexion/extension, lateral bending, and axial rotation from several *in vivo* and *in vitro* studies. A 10 kg box and two 5 kg dumbbells were added in the model with the same size, mass, location, and orientation as those used in the experiments while also considering the hand-load contact reaction forces. Three markers captured the motion trajectories of the hand load during the tasks. Markers were defined in the model precisely as they were placed on the weight during the motion capturing.

For each subject, the model was adjusted in terms of body height, body weight, and segmental lengths according to the subject’s body measures. Distribution of segmental body masses and body fats were also adjusted (Frankenfield et al., 2001; Winter, 2009). Simulations by an AnyBody motion-captured model required the subject-specific kinematic data as input, and consisted of the following two steps: parameter optimization and inverse dynamic. In the first step, the model was adjusted subject-specifically. The segmental lengths were scaled using a linear method through an optimization procedure that minimized the least-square errors for virtual markers on the model according to the position of corresponding experimental reflective markers placed on the subject (Andersen et al., 2010). Besides, muscle strengths were also scaled using length-mass-fat scaling law by taking the body fat percentage into account (Rasmussen et al., 2005). The optimized and scaled model was then used in kinematic analysis to calculate joint angles from an over-determinate kinematic solver.

In the second step, individual joint angles together with the measured GRFs were used as input for the inverse dynamics analysis. In the course of an inverse dynamics simulation, joints and muscle forces were estimated from known kinematics by solving Newton’s equations. As muscles outnumbered the Newton’s equations for a given movement (i.e., joint kinetic redundancy), an optimization algorithm was applied to estimate muscle forces (Damsgaard et al., 2006). In this study, a third-order polynomial objective function minimizing the sum of cubed muscle stresses was employed (Arjmand and Shirazi-Adl, 2006).

### Female-specific Models

To develop female-specific models, the anatomical attributes that vary as a function of sex were taken into account, and the base-models were modified for females. Muscle physiological cross-sectional areas (PCSAs), segmental mass distributions, and body fat percentage were modified based on available *in vivo* data. PCSAs of trunk muscles were taken from (Marras et al., 2001; Stokes et al., 2005) (Table 2). Mass distribution of each segment was calculated from (Shan and Bohn, 2003) based on a regression equation that estimates the segmental mass distribution based on the body mass and height (Table 3). Moreover, body fat percentage for females was calculated using a regression equation (Frankenfield et al., 2001) based on BMI.

**TABLE 2:** PCSAs (cm^2^) of trunk muscles for male and female base-models as well as female-specific model.

Muscle	Male and female base-models	Female-specific model
Multifidus	14.07	9.49
Erector Spinae	27.89	16.14
Quadratus Lumborum	4.41	2.44
Psoas Major	14.63	10.67
Internal Oblique	6.24	6.30
External Oblique	6.24	7.08
Rectus Abdominis	7.80	6.37

**TABLE 3:** Mass distribution of body segments for male and female base-models as well as female-specific model. Values are expressed as the percentage of total body mass (% of BW).

Segments	Male and female base-models	Female-specific model
Head	8.10	7.97
Thorax	21.60	20.25
Lumbar	13.90	12.43
Pelvis	14.20	11.54
Thighs	20.00	26.62
Shanks	9.30	10.24
Feet	2.90	2.60
Upper arms	5.60	4.70
Lower arms	3.20	2.60
Hands	1.20	1.04

A base-model was developed for each male and two models were developed for each female participant to consider the sex differences into account; female base-model (before applying sex-specific parameters) and female-specific model that was a modified version of the base-model according to the abovementioned female-specific parameters. First, both male and female participants were simulated by the base-model for all the tasks then females were simulated with the corresponding developed female-specific model. Total of 360 model simulations (10 male base-models + 10 female base-models + 10 female-specific models times 12 tasks) were carried out in AnyBody.

### Data Analyses

The resulting forces were calculated over the 3 s of the holding period. Global and local trunk muscle forces as well as L5-S1 compressive and shear (resultant of mediolateral and anteroposterior) loads were computed. Statistical analyses were performed in MATLAB R2019b (The Math Work, Inc.). Pearson analysis was used to analyze anthropometric data. Independent (unpaired) student t-test was applied to assess the sex-dependent difference between males and females. A *p*-value < 0.05 was considered statistically significant. Paired student t-test was applied to assess loads predicted by models for females before and after applying the sex-dependent parameters. Pearson correlation analysis was applied to determine the correlation between normalized measured EMG and model predicted muscle activities (muscle force divided by muscle strength).

## Results

### Spinal Loads in Male’s vs. Female’s Base-Models

**FIGURE 3:**
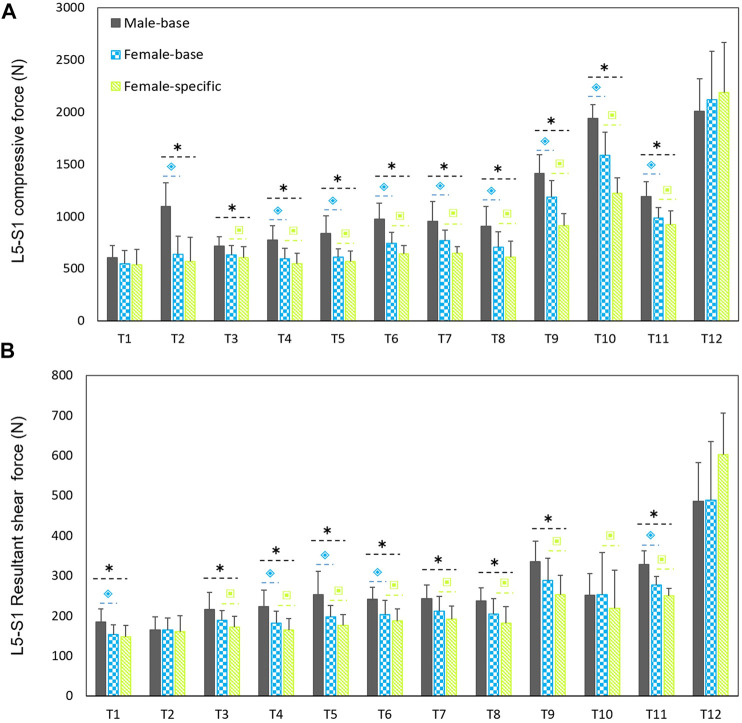
Predicted mean (standard deviations as error bars) absolute L5-S1 compressive **(A)** and shear **(B)** forces by the base-models for both sexes and female-specific models for females. ⌺ indicates a significant difference (*p* < 0.05) between males and females load predicted by base-models. * indicates a significant difference (*p* < 0.05) between loads predicted by male models and female-specific models. ⌺ indicates a significant difference (*p* < 0.05) between loads predicted by female-base models and female-specific models.

**FIGURE 4:**
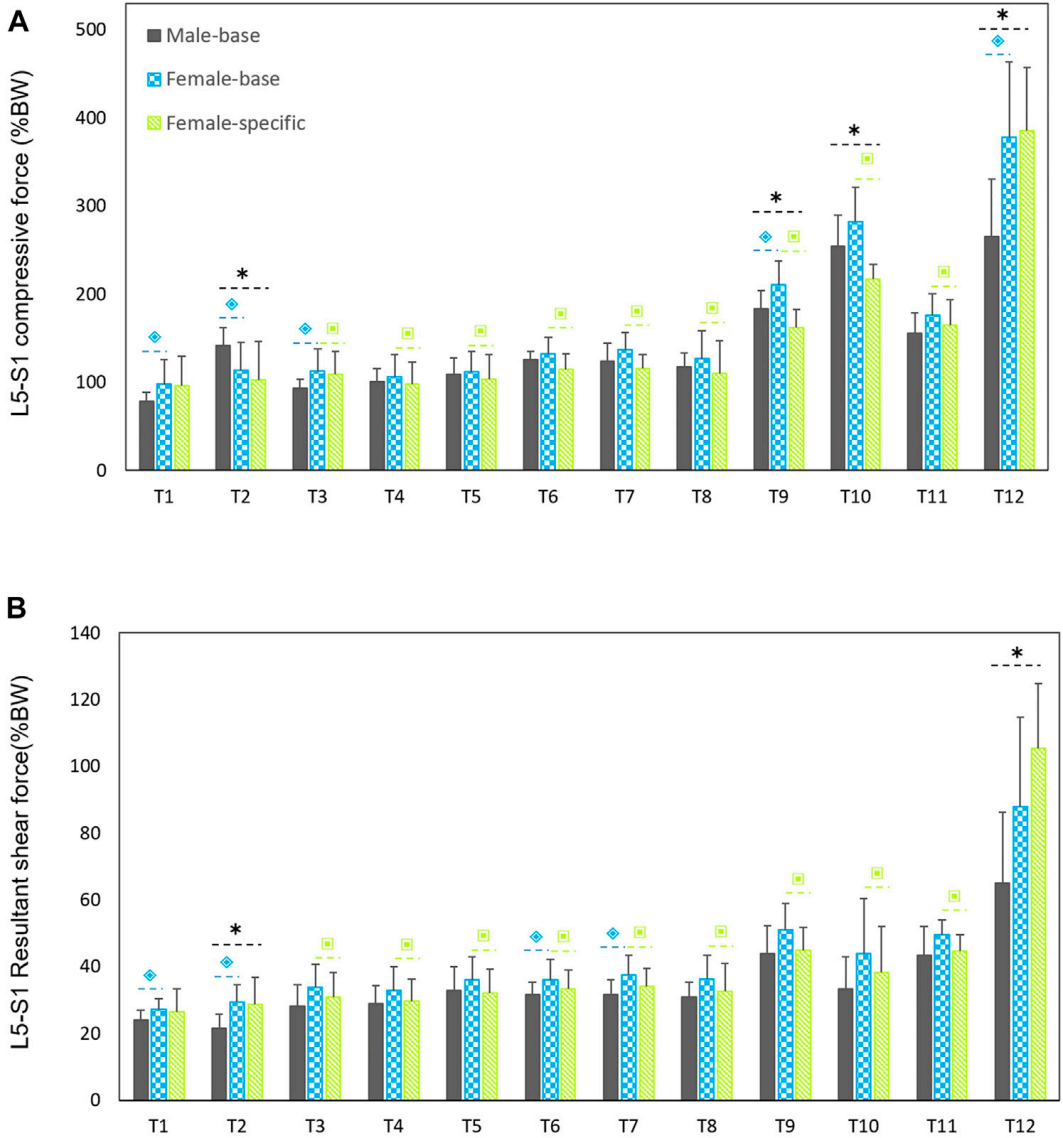
Predicted mean (standard deviations as error bars) L5-S1 compressive **(A)** and shear **(B)** forces normalized to body weight (%BW) by the base-models for both sexes and female-specific models for females. ⌺ indicates a significant difference (*p* < 0.05) between males and females load predicted by base-models. * indicates a significant difference (*p* < 0.05) between loads predicted by male models and female-specific models. ⌺ indicates a significant difference (*p* < 0.05) between loads predicted by female-base models and female-specific models.

Males had considerably larger L5-S1 compressive and resultant shear loads than females in average (25.3% compressive and 14% shear loads) for all the simulated tasks but T12 (Figure 3). The lowest and highest loads, without significant differences between males and females (base-models), were predicted for, respectively, task T1 (upright standing posture) and T12 (trunk axial rotation with 10 kg load in hands). Interestingly, in females, the compressive forces for symmetric lifting tasks in the sagittal plane only slightly varied in T1 through T8 tasks despite the fact that a 10 kg weight was held in hands for some of these activities; i.e., only T9 to T12 tasks caused a substantial load increase as compared to T1 task in females. For males, flexion task (T2) resulted in a significant increase in L5-S1 compressive loads (Figure 3A). When the predicted loads were normalized to the BW, the large differences between the base-models of males and females disappeared and even for some tasks the predicted loads in females became slightly larger than those in males (Figure 4). That is, female base-models predicted, in average (of all tasks), larger compressive (9%) and shear (16.2%) normalized loads than males’ based-models.

### Spinal Loads in Female-Specific Models

On average (of all tasks), the predicted absolute forces by males’ models were considerably larger than the female-specific models (41% compressive and 23.6% shear loads). However, the normalized (to BW) compressive and shear loads in female-specific models were, respectively, 2.4% smaller and 9.4% larger than males in average. Moreover, the predicted loads by the female-specific models were significantly smaller than those predicted by the female based-models except for T12 task (Figure 3) (*p* < 0.05 in most of the simulated tasks). The largest effect of female-specific parameters on the predicted L5-S1 loads was 22.7% reduction in the predicted compressive load (task T9) and 18.6% increase in the predicted shear load (task T12) (Figure 3). Such an effect was in average (all tasks) 11.4 and 9.8% reduction for the predicted compressive and shear loads, respectively.

### Muscle Forces

**FIGURE 5:**
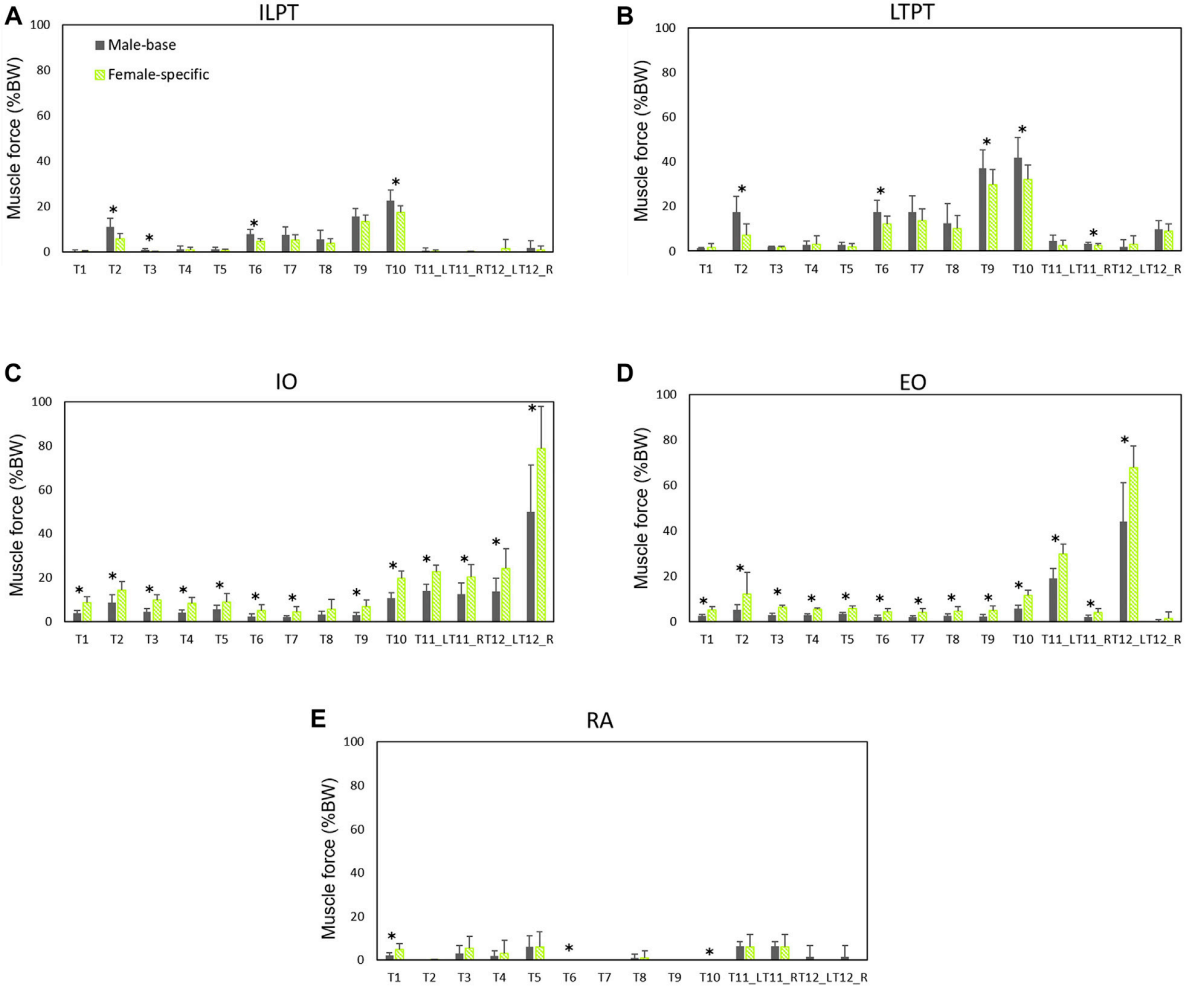
Global muscle forces **(average of left and right)** as predicted by males’ models and female-specific models: **(A)** iliocostalis lumborum pars thoracic (ILPT), **(B)** longissimus thoracis pars thoracic (LTPT), **(C)** internal oblique (IO), **(D)** external oblique (EO), and **(E)** rectus abdominis (RA). * indicates a significant difference (*p* < 0.05) between males and females.

**FIGURE 6:**
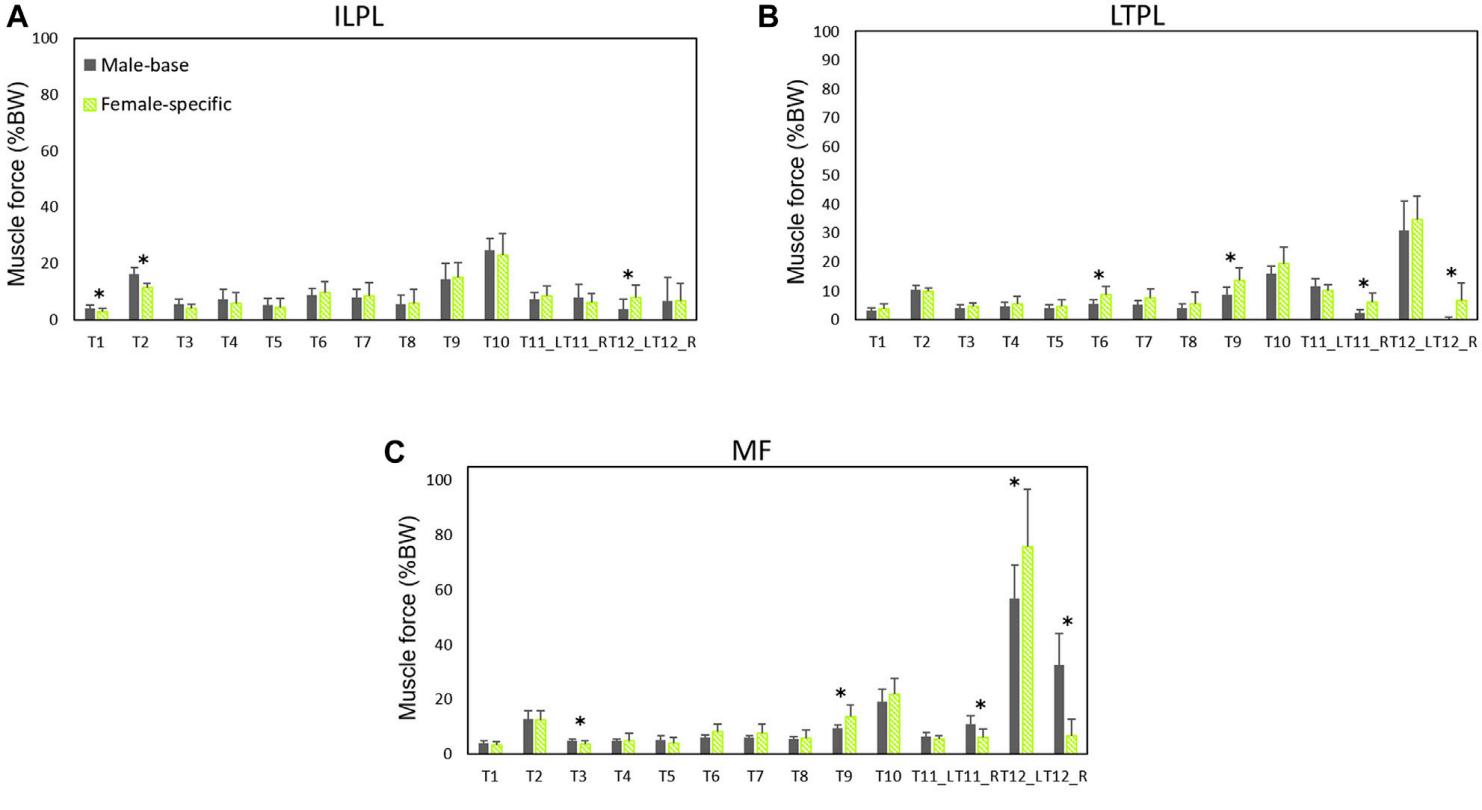
Local muscle forces **(average of left and right)** as predicted by males’ models and female-specific models: **(A)** iliocostalis lumborum pars lumborum (ILPL), **(B)** longissimus thoracis pars lumborum (LTPL), and **(C)** multifidius (MF). * indicates a significant difference (*p* < 0.05) between males and females.

Normalized (to BW) muscle forces predicted by males’ models and female-specific models are shown in Figures 5, 6. Females showed larger oblique muscle forces (*p* < 0.05 in most of the simulated tasks) while males had larger trunk extensor muscle (global) forces during most of the symmetric lifting tasks (*p* < 0.05). The maximum global force for symmetric lifting tasks in both groups was predicted for the longissimus thoracis pars thoracic (LTPT) muscle in task T10 (41.8 %BW for males and 32.2 %BW for females) (Figure 5). In asymmetric tasks, maximum global muscle forces were predicted in T12 for the internal oblique muscle (50 %BW for males and 78 %BW for females) (Figure 5).

For the local muscles, the highest forces for symmetric lifting tasks were predicted in T10 for iliocostalis lumborum (ILPL) muscle (24.8 %BW for males and 23 %BW for females) (Figure 6). For asymmetric lifting tasks, multifidus showed the largest force (56.7 %BW for males and 75.7 %BW for females) (Figure 6).

### Predicted vs. Measured Muscle Activities

The measured EMGs collected from twelve back and abdominal muscles and their corresponding model predicted muscle activities (female base-model, female-specific model, and male base-model) were compared (Table 4). As compared to female base-model, female-specific model improved the correlations for back extensor muscles. The erector spinae muscles (iliocostalis and longissimus) showed a strong correlation (r = 0.72) in females, and a moderate correlation in males (r = 0.57). For abdominal oblique muscles, the correlation was moderate for females (r = 0.50) and weak for males (r = 0.27). For both sexes, internal oblique muscles showed a higher correlation than external oblique muscles. The small measured and predicted activities in rectus abdominis (Figure 5) were poorly correlated for both sexes (Table 4).

**TABLE 4:** Pearson correlations between muscle activities predicted by models and EMG signals recorded from participants’ muscles during lifting tasks. The bolded values indicate a linear relationship between measured and predicted muscle activity (*p* < 0.05).

	Male base-model		Female base-model		Female-specific model	
	r	p-vale	r	p-vale	r	p-vale
multifidus (left)	0.38	0.22	0.35	0.26	0.40	0.20
multifidus (right)	0.38	0.22	0.54	0.07	0.68	**0.02**
Lumbar erector spinae (left)	0.31	0.32	0.35	0.27	0.52	0.08
lumbar erector spinae (right)	0.76	**0.00**	0.47	0.13	0.81	**0.00**
thoracic erector spinae (left)	0.65	**0.02**	0.70	**0.01**	0.81	**0.00**
thoracic erector spinae (right)	0.54	0.07	0.77	**0.00**	0.75	**0.01**
internal oblique (left)	0.67	**0.02**	0.92	**0.00**	0.79	**0.00**
internal oblique (right)	0.02	0.95	0.67	**0.02**	0.66	**0.02**
external oblique (left)	0.36	0.25	0.90	**0.00**	0.90	**0.00**
external oblique (right)	0.03	0.92	-0.26	0.42	-0.38	0.22
rectus abdominis (left)	-0.22	0.50	0.03	0.91	-0.05	0.87
rectus abdominis (right)	-0.27	0.39	-0.24	0.45	-0.30	0.34

## Discussion

The present study aimed to predict spinal loads and trunk muscle forces at the lumbosacral (L5-S1) joint during a number of manual material handling tasks using full-body, subject- and sex-specifics models driven by subject-specific *in vivo* kinematic data. Literature has demonstrated notable sex-dependent differences in joint angles during lifting activities (Plamondon et al., 2017; Sheppard et al., 2016), lumbo-pelvic coordination (Pan et al., 2020; Pries et al., 2015), lifting style (Haddas et al., 2015; Lindbeck and Kjellberg, 2001), muscle cross-sectional area (Marras et al., 2001; Anderson et al., 2012), anthropometry measures, and mass distribution (Shan and Bohn, 2003). These sex-specific parameters influence spinal loads and can explain sex-dependent differences in the predicted spinal loads.

Comparing the predicted L5-S1 loads in females with and without applying sex-specific parameters showed a maximum of ∼23% reduction for the L5-S1 compressive forces in tasks T9 and T10 (Figure 3). Our results showed that sex could significantly affect predicted spinal loads and revealed that the differences in spine loads between males and females were not only a function of body size. In contradiction with earlier findings (Ghezelbash et al., 2016) that showed sex had a small effect on spinal loads, in our study sex-specific parameters for females significantly affected the predicted loads in almost all tasks. On average (all the simulated tasks), the compressive and shear forces were 11.4 and 9.8%, respectively, smaller in female-specific models than female-base models. Ghezelbash et al. (2016), assumed identical body weight, height, and age for their males’ and females’ models and showed that during symmetric lifting activities the effect of sex on spinal loads was small (0.7% for compression and 2.1% for shear). In the present study, however, the effect of sex was found to be much larger (18% for compression and 10.9% for shear) during symmetric lifting tasks (T6-T10). This could be explained by the fact that (Ghezelbash et al., 2016) neglected proper sex/subject-specific kinematics. Moreover, they used kinematics data of unloaded motion (Pries et al., 2015) to simulate lifting tasks while holding external loads in hands influences trunk kinematics (Davis and Marras, 2000; Granata and Sanford, 2000). Altogether, these assumptions in the study of Ghezelbash et al. (2016) may explain their findings as to the small effect of sex on spinal loads. Our findings showed that spinal loads in females and for almost all the simulated tasks except T12 were in average smaller (11.8% for compressive and 9% for shear forces) when the female-specific models were used. Note that the PCSA’s values of back muscles in the base-models were larger (39%) than corresponding values in female-specific models.

Both sexes showed large L5-S1 compressive and shear forces in task T12. Combination of trunk axial rotation and load-handling is a significant risk factor for back injuries. External and internal oblique muscles have been identified as prime trunk rotators. It has been shown that during axial rotation, compared to movements in the coronal or sagittal planes, higher co-contractions are produced in these muscles (Ng et al., 2001), resulting in increased spinal loads (Granata and Marras, 1995). In agreement, in our study lifting a 10 kg hand load while also twisting the trunk (task T12) showed the highest L5-S1 loads and muscle forces of the contralateral external oblique and ipsilateral internal oblique (Figures 3–5). In the female base-models, the PCSAs of oblique abdominal muscles was, in average, 7.2% smaller than PCSAs of female reported in the literature (Marras et al., 2001) that used in female-specific model. This could be the reason for the increase of the predicted spinal loads (7.3% compressive, 18.6% shear) in task T12 (lifting while twisting the back) for females after applying sex-specific parameters. Therefore, it is important to consider sex-specific parameters, especially when simulating tasks with a large trunk axial rotation.

In agreement with previous EMG-assisted biomechanical model (Marras et al., 2002; Marras et al., 2003), and subject-specific kinematics driven models (Ghezelbash et al., 2018), in our study males showed larger absolute compressive (41%) and shear (23.6%) loads. In these studies, however, other confounding parameters such as BW and body height were not controlled. It has been shown that BW markedly affects spinal loads (Ghezelbash et al., 2016; Hajihosseinali et al., 2015), thus larger absolute spinal loads in males could partially be due to their larger body masses. Marras et al. (2002), during two lifting conditions (isolated torso and whole-body free-dynamic), showed that even when differences in body weight were accounted for, sex differences in spine loading persisted. He showed when lifting motions were confined to torso (i.e., having the same lifting style), the sex differences in the spine loading were directly due to the variations in BW. However, when greater kinematics freedom was permitted, females’ spinal loads increased as compared to those in males. According to their findings, it became complicated to relate spinal load differences between males and females to their BW alone. Spinal load differences also are linked to the degree of control required during exertion (Marras et al., 2002; Marras et al., 2003). Females adopt different lifting kinematics in demanding lifting activities. While females perform these tasks by mainly relying on their hips, males rely more on their lumbar spine. The larger motion in females’ hip is attributable to their lower trunk strength. In our study, during task T12 (lifting and twisting the trunk), which is a demanding task, larger absolute and normalized spinal forces were predicted in females, despite their smaller body mass as compared to male participants. However, when the predicted loads were normalized to BW, the difference in spinal loads between males and females almost disappeared (males had 2.4% larger compressive, and 9.4% smaller shear loads than females). By assuming identical BWs in males’ and females’ models, (Ghezelbash et al., 2016) also found small differences in spinal load between both sexes; females had slightly larger (4.7% for compression and 8.7% for shear) loads than males. Moreover, we compared the predicted relative loads for four matched participants [2 males (weight: 61.9 kg, height: 166.3 cm) and 2 females (weight: 60.8 kg, height:165.5 cm)]. Males had, in average, ∼%6 larger relative compressive forces than females thus confirming the general finding of our study.

Our previous *in vivo* study on a large asymptomatic population (141 males and 179 females) indicated that BMI did not affect lumbar range of flexion or spine rhythm (Zander et al., 2018) as long as BMI remains below a threshold of 26 kg/cm^2^. More importantly, another recent study of our group (Ghasemi and Arjmand, 2021) found that BMI had no significant effects on the three-dimensional spine (trunk, lumbar, and pelvis) kinematics of males during various symmetric and asymmetric load-handling activities. Moreover, some studies reported remarkable effects of sex on lifting kinematics (Lindbeck and Kjellberg, 2001; Plamondon et al., 2014). Similarly, significant lumbo-pelvic movement differences between females and males were reported in our previous study (Pries et al., 2015); larger contribution of the pelvis and less trunk flexion in females compared to males. Altogether, these findings indicate that spine kinematics are mainly affected by sex rather than BMI.

It has been shown that during identical lifting activities, females produce higher levels of muscle activities (Marras et al., 2003). In agreement, our models (on average) predicted slightly higher muscle forces in females. Measured and predicted muscle activities showed a stronger correlation for females. A linear correlation between AB predicted muscle activities and measured EMGs for erector spinae muscles has been found during lifting activities at two different heights (r = 0.62 and r = 0.70) (Stambolian et al., 2016). In agreement, our study showed a strong correlation (r = 0.72) in females and moderate (r = 0.57) correlation in males for the erector spinae muscles.

Females on average are smaller in size and have lower muscular strength than males (Lindbeck and Kjellberg, 2001; Plamondon et al., 2014). Significantly smaller muscle PCSAs in females (Anderson et al., 2012; Marras et al., 2001) could, at least partly, be responsible for their smaller muscular strength. Females’ lifting strength ranges between 48 and 70% of that of males (Kumar and Garand, 1992; Marras et al., 2002; Plamondon et al., 2014), and therefore they have lower spine tolerant limits. Sex differences in strength have an impact on their lumbo-pelvic coordination and their muscle activity patterns. Larger contribution of the pelvis in females during lifting tasks might be a compensation mechanism to help them flex less their trunk due to the lower trunk strength capacity in the lumbar region (Marras et al., 2002). Furthermore, females tend to increase their muscle activities to stabilize the trunk and flex it less (Lindbeck and Kjellberg, 2001). Subramaniyam et al. (2019) showed sex-dependent muscle activity patterns during identical lifting tasks. Abdominal coactivities increase spinal stability during lifting (El-Rich et al., 2004), and significantly contribute to spine shear forces (Marras et al., 2002). *In vivo* studies, in agreement with our simulation results, showed that females had more active trunk stabilizer muscles, MF, IO, and EO, during symmetric and asymmetric lifting tasks (Marras et al., 2002; Subramaniyam et al., 2019). Although the higher activity of nonprimary extensor muscles during lifting plays a stabilization role for the trunk by providing greater stiffness, it could adversely increase spinal loads (El-Rich et al., 2004). A comparison of spine loads relative to the tolerance limits indicated that females were 25% closer to their expected tolerance (Marras et al., 2003). Having higher muscular coactivities and smaller strength capacity, cause females to experience greater muscle fatigue and be more vulnerable to muscle strain and injury. This is supported by findings of the epidemiological studies that report higher work-related physical injuries (Hansen et al., 2018) and a higher prevalence of low back pain (Wu et al., 2020) in females than males. Under different lifting conditions in the work environment, female workers also behave differently than males in terms of kinematics and muscle activities (Plamondon et al., 2014). Altogether, and taking into account the sex-specific differences, males and females are to be treated differently while designing their work environments (Lindbeck and Kjellberg, 2001).

This study had some limitations. The BMI of male and female participants was not controlled. Unequal body masses influence absolute spinal loads (Ghezelbash et al., 2016; Hajihosseinali et al., 2015). Although loads normalized to a subject’s BW account for certain anthropometric differences, it would be preferable to consider matched male-female subjects (in terms of BW and BH) when sex-dependent spinal load differences are investigated during identical lifting activities. Soft tissue artifacts are the main source of errors in skin marker-based motion analysis (Benoit et al., 2006; Leardini et al., 2005; Stagni et al., 2005). In order to minimize such errors, a local optimization method (Andersen et al., 2010) was used to update the initial segment lengths and marker locations on the model with respect to the experimental ones. As motion capture data do not provide individual lumbar vertebrae kinematics, a pre-defined 3D lumbar spine rhythm was used to define intervertebral rotations during upper body inclination; the likely inter-individual differences in lumbar spine rhythm were overlooked (Arshad et al., 2016; Pearcy, 1985; Zander et al., 2018). Recorded skin EMGs were limited to select muscles subjected also to the cross-talk issue. As to the model itself, force-length-velocity relationships were neglected. Spinal ligaments and facet articulations were not considered and intervertebral joints were modeled as spherical joints with fixed centers of rotation. The moment arms of muscles were not sex-dependent in the model. AnyBody Modeling System (Damsgaard et al., 2006) uses a general linear scaling approach to adjust the segment-fixed insertion nodes of muscles based on subject’s anthropometric characteristics. This is in accordance with the MRI imaging study (Jorgensen et al., 2001) that showed the distance of the muscles from the spine (e.g., moment arm) depends upon anthropometric characteristics such as torso depth/breadth, body mass, and stature. Finally, while sex-dependent parameters influenced spinal loads, their distinct effects remains to be investigated.

## Conclusion

The present study aimed to predict spinal loads and trunk muscle forces at the lumbosacral (L5-S1) joint during a number of manual material handling tasks using full-body, subject- and sex-specifics models driven by subject-specific *in vivo* kinematic data. Base-models (subject-specific segmental length, muscle architecture, and kinematics data) used for both sexes. For females, female-specific models were also developed by taking into account the female-specific parameters (muscle physiological cross-sectional areas, segmental mass distributions, and body fat percentage). Males showed significantly larger absolute compressive and shear spinal loads than females for almost all the simulated tasks in this study. When the spine loads were normalized to BW, differences between the predicted spinal load for males and females became less pronounced. Female-specific models predicted significantly smaller L5-S1 loads as compared to female base-model. Neglecting sex-specific parameters in musculoskeletal models of the spine could result in overestimation of the spinal loads in females.

## Data Availability

The raw data supporting the conclusion of this article will be made available by the authors, without undue reservation.
